# Robot-assisted sacral tumor resection: a preliminary study

**DOI:** 10.1186/s12891-018-2084-9

**Published:** 2018-06-06

**Authors:** Junqiang Yin, Hui Wu, Jian Tu, Changye Zou, Gang Huang, Xianbiao Xie, Yulong He, Jingnan Shen

**Affiliations:** 1 Department of Musculoskeletal Oncology, First Affiliated Hospital of Sun Yat-Sen University, Guangzhou, 510080 China; 2 Department of gastrointestinal surgery, First Affiliated Hospital of Sun Yat-Sen University, Guangzhou, 510080 China

**Keywords:** Robot-assisted surgery, Da Vinci surgical system, Sacral tumor

## Abstract

**Background:**

Few studies have been done on robot-assisted sacral surgery. This study aims to evaluate the outcomes of seven patients with benign sacral or presacral tumors treated with a robotic surgical system at a single center.

**Methods:**

All patients with benign sacral or presacral tumors who underwent transperitoneal resection (between June 2015 and June 2016) using the da Vinci Si HD robotic surgical system (Intuitive Surgical Inc.) were included in this retrospective study.

**Results:**

Seven patients with a mean age of 43.8 years (range: 22- 62 years) were included in this study. The operation time ranged from 60 to 335 min. Five out of these seven patients with presacral tumor underwent complete tumor resection by the da Vinci robotic surgical system, with a median blood loss of 52 ml. The other patients underwent excision of the presacral tumor by the da Vinci robotic surgical system, followed by a posterior approach, with a median blood loss of 675 ml. The histological diagnosis was schwannoma of the sacral nerve in five cases (71.5%). The other two cases were chordoma and solitary fibroma of the sacrum, respectively. No perioperative or postoperative complications were encountered. The average hospitalization stay was 5.7 days. No recurrences were found at follow-up 24 to 31 months later.

**Conclusion:**

Robot-assisted minimally invasive sacral surgery can provide precise dissection of the tissue under a perfect view. It is a technically feasible procedure that is associated with minimal blood loss, fewer injuries and short hospitalization. It is particularly suitable for presacral benign tumors.

## Background

Primary tumors originated from sacrum or retraperitioneal area are rare. Most of them are benign or low-grade malignancies, such as chondrosarcoma and schwannoma [1, 2]. The resection of the tumor was one of the most important treatments for primary malignant sacral tumors, which could be performed using anterior, posterior, lateral and combined surgical approaches. The treatment of primary sacral tumors can be challenging because of the complex surrounding anatomical structures and the large size of the tumor [3]. The da Vinci surgical system is widely used in gastrointestinal surgery, urology and gynecological surgery, as it can provide the following advantages [4–6]. It can provide 3D vision and visual magnification (up to 15- times), which can promote more precise resection. Additionally, the da Vinci surgical system consists of three or four robotic arms, mimicking human wrist movements, with a high degree of freedom. However, there is no report about the performance of the da Vinci surgical system in the resection of sacral tumor.

We aimed to investigate the feasibility and efficiency of the da Vinci surgical system in the resection of sacral tumors.

## Methods

### Patients

Patients with a sacral or presacral tumor who were treated using da Vinci surgical system at the First Affiliated Hospital of Sun Yat-sen University between June 2015 and January 2016 were enrolled in this retrospective study. Written informed consent to participate in the study was obtained from participants. This study was approved by the Institutional Ethical Board of the First Affiliated Hospital of Sun Yat-sen University.

The criteria for case inclusion were as follows: (1) presence of a tumor located in sacral or pre-sacral area; (2) operated by da Vinci surgical system. Patients with previous sacral or presacral surgery history were excluded following a discussion. Patients without completed follow-up information were excluded.

### Surgical treatment procedure

Clinical assessment, including a thorough history, physical examination, digital rectal examination, routine blood tests, chest X-ray, computerized tomography, magnetic resonance imaging of the pelvis and needle biopsy, was completed for all patients before robotic surgery.

**Fig. 1:**
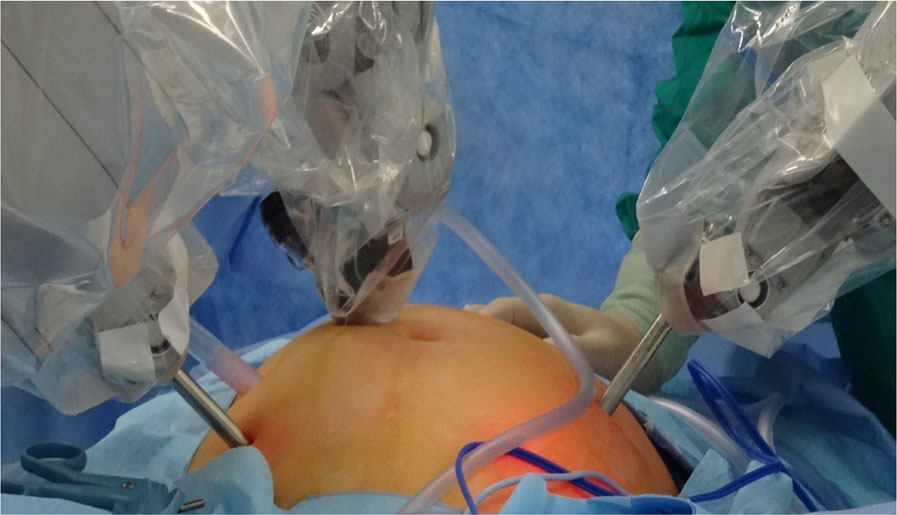
surgical position and trocars placement

With patients in the supine position, general anesthesia was administered for patients (Fig. 1). A small incision was made on the right side 3 cm above the umbilicus to place a 12-mm trocar for the camera. The other three 8-cm trocars were placed as shown in the Fig. 1. The pressure of the pneumoperitoneum was maintained at 15 mmHg. The posterior peritoneum was dissected to expose the tumor in the presacral area, according to the location of the tumor. The anterior approach allowed the surgeon to identify and protect the internal iliac vessels, ureter and rectum and determine the proper plane of the resection. The tumor was resected and removed only using the anterior approach if the tumor size was not too large or the location was proper. However, the posterior approach was then used for tumor removal and spino-pelvic reconstruction if necessary.

### Outcome assessment

Data on peri-operative outcomes, operation time, blood loss, time to eating liquid, hospitalization, cost and complications were recorded. Data on post-operative outcomes, the function assessment (motor and sensory function assessment), tumor recurrence and metastasis (X ray and MRI) were examined every 3 months for Years 1 and 2, every 6 months for Years 3 to 5 and yearly thereafter, as our previous studies described [7].

## Result

**Fig. 2:**
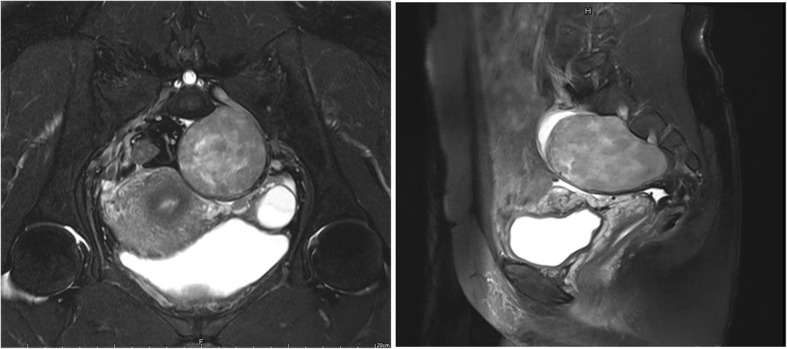
Patient with small presarcral tumor underwent complete tumor resection by the da Vinci robotic surgical system

**Fig. 3:**
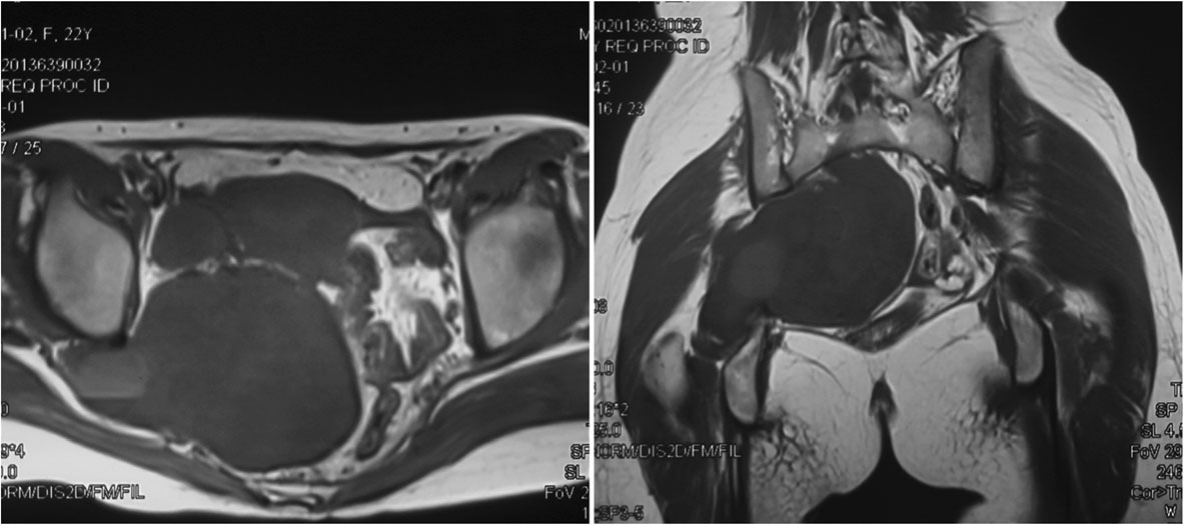
Patient with large presarcral tumor underwent excision of the presacral tumor by the da Vinci robotic surgical system, followed by a posterior approach

Seven patients were included in this study and successfully underwent operation suing the da Vinci surgical system (Table 1). Five of the patients with presacral tumor were operated using only anterior approach, as the tumor sizes were 37.7 cm^3^, 113.0cm^3^, 39.3cm^3^, 141.4cm^3^ and 96.3 cm^3^, and the locations were proper (Fig. 2). The operation time of these 5 patients were 60 min, 90 min, 100 min, 80 min and 90 min, respectively, and the blood loss amounts were 50 ml, 100 ml, 30 ml, 50 ml and 30 ml respectively. The fourth patient with schwannoma was operated on using the anterior and posterior approaches, as the size was 433.4cm^3^. Additionally, it was adjacent to the greater sciatic foramen and sciatic nerve (Fig. 3). The posterior approach was taken to ensure the safe separation of the tumor from sciatic nerve and vessels accompanied. The operation time of this patient was 200 min, and the blood loss amount was 600 ml. The fifth patient, with chordoma and sacrum involved, was operated on using anterior and posterior approaches. The ovarian artery was closely related to the tumor, so the da Vinci surgical system was employed to separate this vessel and ligature the presacral vessels. The operation time of this patient was 335 min and the blood loss amount was 700 ml.

**Table 1:** Clinical characteristic of patients

Case	Sex	Age- ranges	Pre-operative diagnosis	Tumor size (cm^3^)^a^	Adjacent tissues	Operative time (min)	Blood loss (ml)	Post-operative diagnosis	Hospitalization (days)
1	F	45-55	Schwannoma	37.7	iliac vessel	60	50	Schwannoma	3
2	F	40-50	Schwannoma	113.0	iliac vessel	90	100	Schwannoma	9
3	F	60-70	Solitary fibroma	39.3	superior rectal artery	100	30	Solitary fibroma	5
4	F	20-30	Schwannoma	433.4	intestine	200	600	Schwannoma	5
5	F	50-60	Chordoma	194.2	ovarian artery	335	750	Chordoma	10
6	F	20-30	Schwannoma	141.4	ureter	80	50	Schwannoma	4
7	M	45-55	Schwannoma	96.3	iliac vessel	90	30	Schwannoma	4

^a^Tumor size = (π/6) × height × width ×depth

No perioperative complications were found among these seven patients. No blood transfusions were administered to these patients during perioperative and postoperative period. The average hospitalization date was 5.7 days, with an average cost of 61,000 yuan (RMB). There were no fever, wound infestion, secondary haemorrage, ileus and leakage among these seven patients. No patient was lost of follow up in this study. No motor or sensory dysfunction of lower extremity was assessed in these patients. There has no evidence of recurrence during the follow-up period of 24 to 31 months after surgery.

## Discussion

The incidence of sacral tumor is low. It is relatively hard for surgeons to accumulate much experience in the resection of sacral tumors. Therefore, it is of great importance to summarize the surgical outcomes of new technology in the treatment of sacral tumor. The majority of sacral tumors are primary, including chordoma, schwannoma and giant cell tumor. Surgical treatment remains as the optimal choice for primary malignant sacral tumors. As for benign or low-malignancy sacral tumors when aggravating symptoms or enlargement of tumors occurs during the clinical follow-up, surgery should be taken into consideration However, the complicated anatomical structure, narrow space for operation in the pelvis and abundant presacral vessels make the treatment of sacral tumors extremely challenging [8–10].

In our center, patients with a large mass in the sacrum usually receive hypogastric artery ligation and isolation using the anterior approach, followed by the excision of the tumor through posterior approach. This strategy could provide enough margins for excision and safety related for bleeding from tumor. However, it would increase the duration of the operation and surgical stress for patients. Several studies have reported the use of a laparoscope to ligate the hypogastric artery and isolate the presacral mass [11–13]. The magnifying function of the laparoscope allows for an en bloc resection without damaging the tumor. But, it is still inconvenient for surgeons to separate related tissue and ligate blood vessels, as laparoscope arms are lack of flexibility in the narrow and deep cavity. The robotic surgical system could provide visual magnification and a high degree of freedom for surgeons. However, no studies have reported the use of the robotic surgical system in the excision of sacral and presacral tumor.

The da Vinci surgical system is widely used in gastrointestinal surgery, urology and gynecological surgery. Therefore, we tried to utilize the robotic surgical system in the treatment of sacral tumor.

According to our experience, the use of the da Vinci surgical system in the treatment of sacral tumors, especially presacral benign tumors, is feasible. We chose the supine position with four trocars to build the robotic surgical system. As these five patients were female, the suspension of uterus was taken to expand the field of vision. Three of the five patients underwent total tumor resection using the robotic surgical system. The average operation time was 83.3 min, with blood loss amount of 60 ml for these three patients. This cost less average operative time and less bold loss compared with patients with presacral schwannoma received laparoscopic surgery [14]. The blood loss was much less than that associated with open surgery [15]. Additionally, it could shorten the recovery time for patients. For the other two patients, the robotic surgical system was used to separate organs close to the tumor, which made the resection of the tumor safe through a posterior approach.

Additionally, a multidisciplinary team including surgeons from musculoskeletal oncology, gastrointestinal and urological departments wan strongly recommended to perform robot-assisted surgery for patients with sacral or presacral tumors. However, this study still contained some limitations. The major one was that it was a retrospective study with limitation of enrolled patient number. It restricted the power of our analysis and conclusion. The robot-assisted minimally invasive sacral surgery should be further studies in prospective cohort with more patients included.

## Conclusion

Robot-assisted minimally invasive sacral surgery is a technically feasible and efficent procedure that is associated with minimal blood loss, fewer injuries and short hospitalization. It is particularly suitable for presacral benign tumors.
